# Sustained peripheral immune hyper-reactivity (SPIHR): an enduring biomarker of altered inflammatory responses in adult rats after perinatal brain injury

**DOI:** 10.1186/s12974-021-02291-z

**Published:** 2021-10-19

**Authors:** Yuma Kitase, Eric M. Chin, Sindhu Ramachandra, Christopher Burkhardt, Nethra K. Madurai, Colleen Lenz, Alexander H. Hoon, Shenandoah Robinson, Lauren L. Jantzie

**Affiliations:** 1 Division of Neonatal-Perinatal Medicine, Department of Pediatrics, Johns Hopkins University School of Medicine, 600 N. Wolfe Street, CMSC Building, 6-104A, Baltimore, MD USA; 2 Department of Neurology and Developmental Medicine, Kennedy Krieger Institute, Baltimore, MD USA; 3 Division of Pediatric Neurosurgery, Department of Neurosurgery, Johns Hopkins University School of Medicine, Baltimore, MD USA; 4 Department of Neurology, Johns Hopkins University School of Medicine, Baltimore, MD USA

**Keywords:** Lymphocyte, Cytokine, Immune modulation, Immune priming, Cerebral palsy, Tumor necrosis alpha, CXCL1, Chorioamnionitis, Peripheral blood mononuclear cell

## Abstract

**Background:**

Chorioamnionitis (CHORIO) is a principal risk factor for preterm birth and is the most common pathological abnormality found in the placentae of preterm infants. CHORIO has a multitude of effects on the maternal–placental–fetal axis including profound inflammation. Cumulatively, these changes trigger injury in the developing immune and central nervous systems, thereby increasing susceptibility to chronic sequelae later in life. Despite this and reports of neural–immune changes in children with cerebral palsy, the extent and chronicity of the peripheral immune and neuroinflammatory changes secondary to CHORIO has not been fully characterized.

**Methods:**

We examined the persistence and time course of peripheral immune hyper-reactivity in an established and translational model of perinatal brain injury (PBI) secondary to CHORIO. Pregnant Sprague–Dawley rats underwent laparotomy on embryonic day 18 (E18, preterm equivalent). Uterine arteries were occluded for 60 min, followed by intra-amniotic injection of lipopolysaccharide (LPS). Serum and peripheral blood mononuclear cells (PBMCs) were collected at young adult (postnatal day P60) and middle-aged equivalents (P120). Serum and PBMCs secretome chemokines and cytokines were assayed using multiplex electrochemiluminescent immunoassay. Multiparameter flow cytometry was performed to interrogate immune cell populations.

**Results:**

Serum levels of interleukin-1β (IL-1β), IL-5, IL-6, C–X–C Motif Chemokine Ligand 1 (CXCL1), tumor necrosis factor-α (TNF-α), and C–C motif chemokine ligand 2/monocyte chemoattractant protein-1 (CCL2/MCP-1) were significantly higher in CHORIO animals compared to sham controls at P60. Notably, CHORIO PBMCs were primed. Specifically, they were hyper-reactive and secreted more inflammatory mediators both at baseline and when stimulated in vitro. While serum levels of cytokines normalized by P120, PBMCs remained primed, and hyper-reactive with a robust pro-inflammatory secretome concomitant with a persistent change in multiple T cell populations in CHORIO animals.

**Conclusions:**

The data indicate that an in utero inflammatory insult leads to neural–immune changes that persist through adulthood, thereby conferring vulnerability to brain and immune system injury throughout the lifespan. This unique molecular and cellular immune signature including sustained peripheral immune hyper-reactivity (SPIHR) and immune cell priming may be a viable biomarker of altered inflammatory responses following in utero insults and advances our understanding of the neuroinflammatory cascade that leads to perinatal brain injury and later neurodevelopmental disorders, including cerebral palsy.

## Introduction

Globally, prematurity is a major cause of infant mortality and long-term disability in children [1, 2]. Preterm birth is a principal cause of neonatal morbidity and mortality, accounting for 70% of neonatal deaths [3]. As perinatal care has improved in recent years with modern medical advances in Neonatal Intensive Care Units (NICUs), the survival rate of preterm infants at earlier gestational ages has increased [4, 5]. Accordingly, increased survival of extremely preterm infants is concomitant with an increased number of children who develop severe acute and chronic morbidities, including periventricular leukomalacia (PVL), intraventricular hemorrhage (IVH), necrotizing enterocolitis (NEC), chronic lung disease, severe visual impairment, hearing impairment, cerebral palsy (CP), and cognitive disorders in childhood [6, 7]. Morbidity is inversely proportional to gestational age and preterm infants are at risk for perinatal brain injury (PBI) and its long-term sequelae. Specifically, preterm infants are at risk for developing CP, and many preterm infants develop co-existing cognitive, behavioral disorders, and psychiatric conditions [8–11], including anxiety, deficits of attention and inhibitory control [12–14].

A major cause of preterm birth is chorioamnionitis (CHORIO) [15]. Indeed, CHORIO is the most common pathologic abnormality found in placentae from very preterm infants [16–18]. CHORIO is a common etiology of in utero inflammation and is known to elicit a fetal inflammatory response syndrome (FIRS) via the maternal–placental–fetal axis [19–21]. CHORIO can be defined by clinical and histopathological criteria and separated into maternal and fetal inflammatory components [22]. Specifically, CHORIO refers to inflammation of the chorioamniotic membranes [22]. Though inflammation can occur under sterile conditions [23, 24], infectious pathogenesis predominantly occurs via an ascending infection from the lower genital tract during pregnancy [22]. While acute chorioamnionitis has a broad clinical diagnosis, a more definitive histopathologic diagnosis exists.

CHORIO is sub-classified histologically by the amount and distribution of inflammatory infiltrate, including neutrophils, within the chorion, amnion and umbilical compartments [25, 26]. Concomitant with profound CHORIO-induced inflammation is an effect on placental circulation, and a degree of placental hypoxia–ischemia [27]. Placental cells stressed in CHORIO release danger associated molecular signals (DAMPS) that activate Toll-like receptors (TLRs) and induce cytokine production and neutrophil recruitment [22, 28]. The associated downstream signal transduction, including nuclear factor-kappa B (NF-kB) transcription factor activation are central to inflammatory regulation the production and release of pro-inflammatory cytokines such as interleukin 6 (IL-6), interleukin 1β (IL-1β), and tumor necrosis factor α (TNFα) [29]. These cytokines, especially IL-1β, propagate the expression of chemokines such as C–X–C motif chemokine ligand 1 (CXCL1), which is a chemoattractant for neutrophils [30]. Collectively, these chemokines recruit neutrophils, monocytes, and macrophages from the maternal circulation to propagate intrauterine inflammation.[19, 22, 31].Cumulatively, this placental dysfunction affects the entire maternal–placental–fetal axis, promoting inflammation, increasing inflammatory cellular and molecular trafficking to the developing brain and precipitates neural–immune injury [27]. Previous studies support the critical role that the placental–fetal brain axis plays in neurological development, with alterations or disruptions in this axis leading to brain injury [32–35]. Changes in the uterine microenvironment and subsequent perinatal events trigger injury to the developing immune system. The abnormal developing immune system impacts central nervous system (CNS) development, and precipitates PBI and increasing susceptibility to chronic sequelae later in life [36]. Notably, placental inflammation and neuroinflammation are common features in preterm infants and those with subsequent brain injury [37].

A significant connection between the placenta and the developing brain is the fetal circulation. Depending on severity, placental inflammation can pass through the umbilical cord and bloodstream to catalyze a FIRS. FIRS is defined by elevated umbilical cord plasma cytokines, including IL-6 and funisitis [38] due to direct contact with infected amniotic fluid or the migration of inflammatory cells from the placental circulation [39–41]. After delivery, this can develop into systemic inflammatory response syndrome (SIRS) in the neonate [42, 43]. Neonates with CHORIO have been shown to have elevated c-reactive protein, myeloperoxidase, CCL-2 (MCP-1), matrix metalloprotease 9, IL-1β, IL-6, TNFα, and C–C motif chemokine ligand 5 (CCL5) in their blood, consistent with monocyte, macrophage, neutrophil and T-cell recruitment [44, 45]. Notably, FIRS and SIRS are both defined by elevated levels of pro-inflammatory cytokines and activation of immune cells that alter neuro-immune crosstalk and increase risk of later neurodevelopmental disorders in preterm infants [46, 47]. Increased circulating inflammatory cytokines can pass through the developing blood–brain barrier and trigger neuroinflammatory responses through activation of microglia and astrocytes [48, 49]. The risk of brain injury is increased in infants who have persistent and/or recurrent elevations of pro-inflammatory proteins [50]. Specifically, the increasing breadth of early neonatal inflammation, quantified by the number of protein elevations or the number of functional protein classes elevated, is associated with increased structural and functional brain injury [50–55]. Similarly, the role of inflammatory cytokines in the pathophysiology of impaired white matter development is well defined in children with cerebral palsy and cognitive delay [56].

As the immune system develops along a similar trajectory as the CNS [57], previous clinical studies have looked at immune cells in addition to circulating inflammatory proteins in survivors of preterm birth. Former preterm children with CP at school age have significantly higher levels of TNF-α in the supernatants of peripheral blood mononuclear cells (PBMCs) before and after lipopolysaccharide (LPS) stimulation compared to peer preterm children without CP [58]. In support of this, we have shown in our translational model of CP that PBMCs isolated from rats on postnatal day 7 (P7, term-equivalent age) and P21 (toddler-equivalent age) are hyper-reactive at baseline and in response to a secondary inflammatory stimulus [59]. These findings are concomitant with acute elevations in pro-inflammatory cytokines in placenta, fetal circulation, and brain, structural and microstructural brain injury as seen on magnetic resonance imaging, as well as a spastic-like gait in adult rats with deficits in executive function [59–64]. Despite these prior clinical or preclinical findings, the chronicity and time course of PBMC alterations in children with CP or perinatal brain injury secondary to CHORIO has not been established. This knowledge is necessary to discover the cellular and molecular mechanisms of injury and to define risk of later sequelae in this patient population, including alterations in the maturation of myelination in adolescence and the development of chronic pain in early adulthood. Thus, the goal of the present investigation was to ascertain the time course of sustained peripheral immune hyper-reactivity (SPIHR) and the persistence of peripheral inflammation in a translational rat model of CP secondary to CHORIO. We hypothesized that PBMCs would be chronically primed in adulthood commensurate with persistent immune activation and a diverse inflammatory secretome throughout the lifespan of rats with CP. We posit leukocyte reactivity following perinatal brain injury may be an important biomarker of perinatal CNS insult and inflammatory activation.

## Methods

### Animals

Sprague Dawley rat dams and litters were maintained in a temperature, and humidity-controlled facility with food and water available ad libitum. Animals were subject to 12-h (h) dark/light cycle, with lights on at 0800 h. All experiments were performed in strict accordance with protocols approved by the Institutional Animal Care and Use Committee (IACUC) at the Johns Hopkins University School of Medicine. Protocols were developed and performed consistent with National Research Council and ARRIVE guidelines [65].

### In utero chorioamnionitis (CHORIO)

Pregnant Sprague Dawley rats underwent abdominal laparotomy on embryonic day 18 (E18) as previously reported [59–64, 66–69]. Pregnant rats were induced with 3.0% isoflurane. Via laparotomy, the uterus was externalized, and the uterine arteries were temporarily occluded with aneurism clips to induce transient placental insufficiency. After 60 min, clips were removed and intra-amniotic injection of lipopolysaccharide (LPS 0111: B4, 4 μg/sac; Sigma-Aldrich, St. Louis, MO, USA) was administered to each sac, and the laparotomy was closed. The rat pups were born at term on embryonic day 22 (E22). Sham animals had a laparotomy (no uterine artery occlusion nor LPS) with equivalent duration of anesthesia. For each experiment described, and data represent true *n* (individual rats) from at least 3 different dams per condition. Male and female offspring were used in every outcome measure.

### Serum collection

Blood was taken from each rat for serum analyses. Whole blood was centrifuged at 6,000 g for 15 min. Serum was then removed and stored at -80° C for later analysis. Care was taken to avoid freeze–thaw cycles.

### Peripheral blood mononuclear cell (PBMC) isolation

PBMCs, defined as any blood cell with a round nucleus (i.e., a lymphocyte, a monocyte, or a macrophage), were isolated as previously described [58, 59, 70]. Specifically, on postnatal day 60 (P60, young adult) and P120 (middle-age adult), venous blood was collected from the right atrium. PBMCs were isolated by Ficoll gradient isolation [58]. Equal amounts of peripheral blood were mixed with RPMI 1640 medium (Gibco, Waltham, MA, USA), and gently placed over 3 mL of Ficoll Paque Plus (17-1440-02, GE Healthcare, Chicago, IL, USA) pre-filled in sterile 15 mL conical tubes. After centrifuging at 400*g* for 30 min at room temperature, PBMCs were harvested, resuspended and washed. Cells were counted using a Countess™ II Automated Cell Counter (Thermo Fisher Scientific). PBMCs were plated at a density of 1 × 10^6^ cells/mL in RPMI medium with 10% FBS. Three replicates were plated for each sample on a 3.5 cm petri dish.

### PBMC stimulation with lipopolysaccharide (LPS)

After plating, 100 ng/ml of LPS or vehicle was added to each well and PBMCs were allowed to incubate for 3 or 24 h as previously described [58, 59, 70]. Upon conclusion of incubation, media were collected and centrifuged at 500*g* for 10 min. Cells and supernatant were subsequently stored separately and stored at − 80 °C until biochemical analysis.

### Multiplex electrochemiluminescent immunoassay (MECI)

A secreted cytokine and chemokine profile analysis was performed on serum or supernatants from cultured PBMCs using a V-PLEX Pro-inflammatory Panel (K15059D, Meso Scale Diagnostics, Rockville, MD, USA) for the detection of interferon gamma (IFN-γ), IL-1β, IL-4, IL-5, IL-6, IL-10, IL-13, CXCL1, and TNF-α. A separate kit was used for the detection of Monocyte Chemoattractant Protein-1 (MCP-1)/ C–C motif chemokine ligand 2 (CCL2) (R-PLEX Rat F231D, Meso Scale Diagnostics, Rockville, MD, USA). In accordance with the manufacturer's specifications and as previously described [62, 70–72], serum or culture supernatant was diluted to 1:4 and then loaded in duplicate with the prepared standard on to a blocked and washed multi-spot 96-well plate. After a series of washes and incubations with antibody detection solution, the plates were washed and loaded with read buffer and then read with a Quickplex SQ 120. Consistent with the standard in the field, samples reading below the detectable limits or with a coefficient of variation greater than 25% in an individual assay were removed from further analyses [59, 62, 70, 72]. Both the pro-inflammatory and CCL2 panels performed with less than 10% variability between runs.

### Flow cytometry

Whole cerebrum at P120 was digested according to the Adult dissociation kit and Miltenyi gentleMACS^TM^ protocol for single-cell suspension [73]. Briefly, the extracellular matrix was enzymatically and mechanically digested using the kit components. Following digestion, cell suspensions were passed sequentially through 70-μm cell filters. After the dissociation, the debris removal solution was used for the elimination of debris, and the red blood cell removal solution was used for the removal of erythrocytes. PBMCs were isolated by Ficoll gradient isolation [58], then eBioscience^TM^ 1 × Red Blood Cell Lysis Buffer (Invitrogen) was used for the removal of erythrocytes. Live cells were counted on a Countess^TM^ II Automated Cell Counter (Thermo Fisher Scientific). Subsequently, 1 × 10^6^ live cells were incubated with a saturating solution of Fc block followed by staining with fluorochrome-conjugated viability dyes and antibodies against: CD45 (Clone OX1; eBiosciences, Waltham, MA), CD11b/c (Clone OX42; eBiosciences, Waltham, MA), Ly6G (Clone RB6-8C5, AbCam, Cambridge, MA), CXCR2 (Clone 866,614, R&D, Minneapolis, MN), CD3 (Clone 1F4; BD Biosciences, San Jose, CA), CD4 (W3/25; Biolegend, San Diego, CA), and CD25 (OX-39; BD Biosciences, San Jose, CA). Data were acquired using the BD LSR-II flow cytometer (BD Biosciences, San Jose, CA) and analyzed using FlowJo software v.10.7.1 (FlowJo LLC, Ashland, OR).

### Statistical analyses

Data are represented as mean ± standard error of the mean (SEM). Parametric statistical differences between 2 groups were compared with Student’s *t* test using Prism 9.1.0. *p* < 0.05 was considered statistically significant.

## Results

### Peripheral inflammation after in utero insult persists into adulthood

**Fig. 1:**
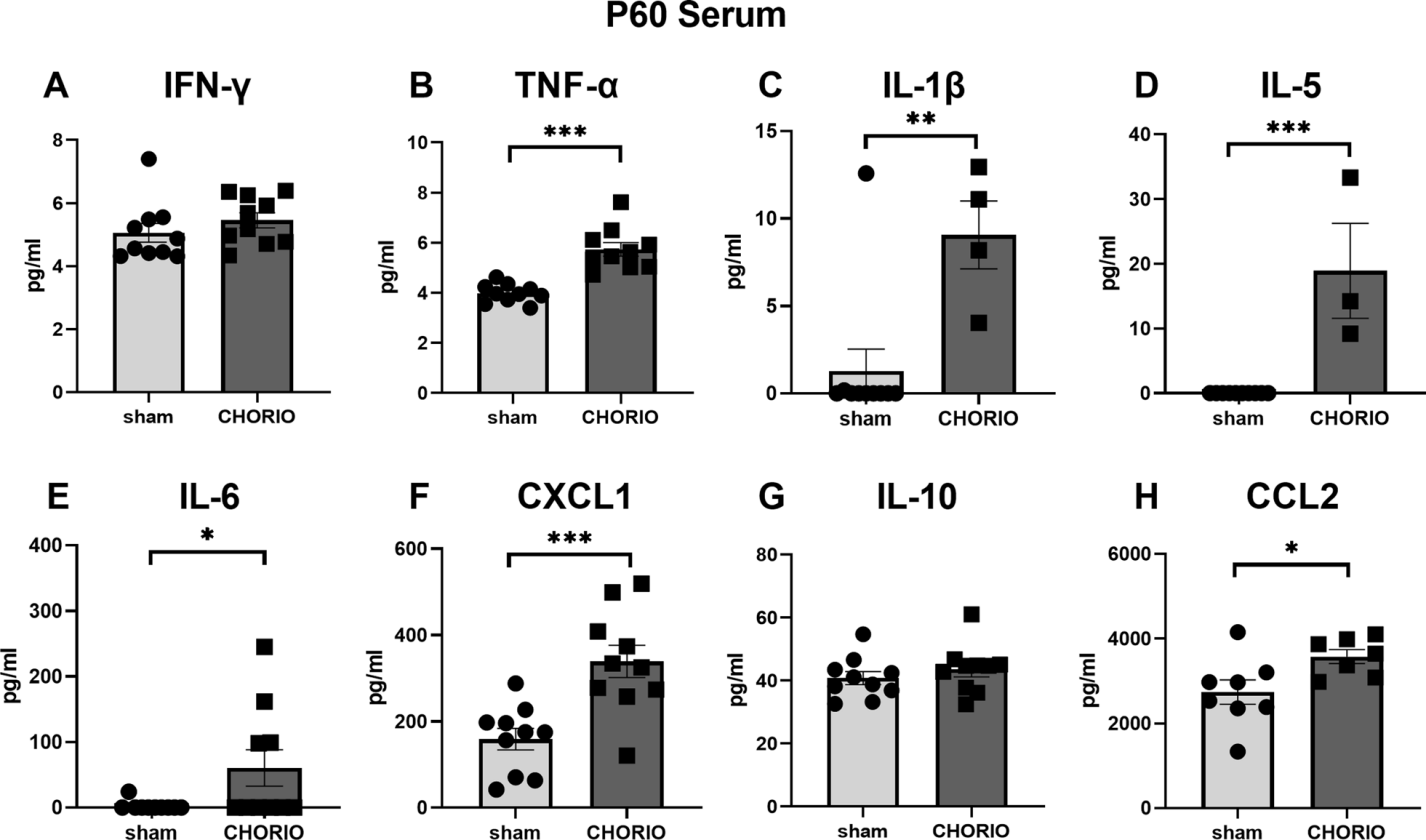
Serum cytokine and chemokine levels in CHORIO and sham rats on P60. Rats with CHORIO have significantly higher TNF-α, IL-1β, IL-5, IL-6, CXCL1, and CCL2 compared to controls. (^*^p < 0.05, ^**^p < 0.01, ^***^p < 0.001)

**Fig. 2:**
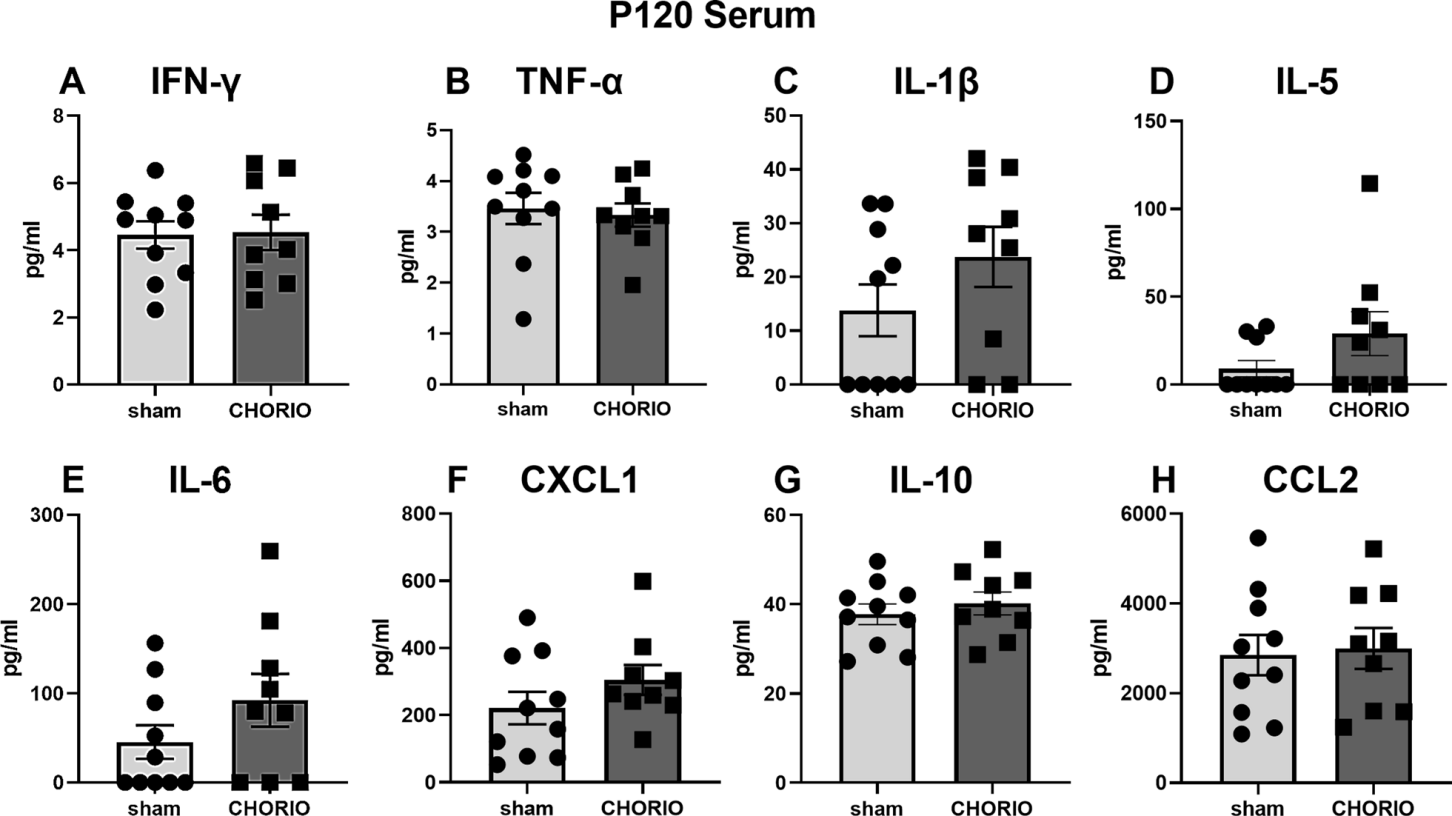
Serum cytokine and chemokine levels in CHORIO and sham animals at P120. No significant differences were observed between CHORIO and sham controls

We began our assessment of the time course of inflammation, by measuring pro- and anti-inflammatory cytokines in serum (*n* = 10/group). Young adult (P60) rats exposed to CHORIO had no significant difference in IFN-γ (sham: 5.06 ± 0.30 pg/ml, CHORIO: 5.46 ± 0.24 pg/ml, *t*-test, *p* = 0.31), but had significantly higher IL-1β (sham: 1.28 ± 1.26 pg/ml, CHORIO: 9.07 ± 1.94 pg/ml, *t*-test, *p* < 0.01), IL-5 (sham: 0.0 ± 0.0 pg/ml, CHORIO: 18.9 ± 7.3 pg/ml, *t*-test, *p* < 0.001), IL-6 (sham: 2.4 ± 2.4 pg/ml, CHORIO: 151.0 ± 34.7 pg/ml, *t*-test, *p* < 0.05), CXCL1 (sham: 158.9 ± 24.8 pg/ml, CHORIO: 338.8 ± 37.5 pg/ml, *t*-test, *p* < 0.001), TNF-α (sham: 3.99 ± 0.12 pg/ml, CHORIO: 5.74 ± 0.27 pg/ml, *t*-test, *p* < 0.001), and CCL2 (sham: 2738 ± 287 pg/ml, CHORIO: 3576 ± 167 pg/ml, *t*-test, *p* < 0.05) (Fig. 1). Interestingly, despite this robust peripheral inflammatory signature at P60, this inflammatory cytokine signature resolved with age. There was no evidence of hypercytokinemia and no significant differences in serum cytokine levels observed between CHORIO and sham rats in older rats at P120 (Fig. 2).

### CHORIO induces a chronic and sustained peripheral immune hyper-reactivity (SPIHR)

**Fig. 3:**
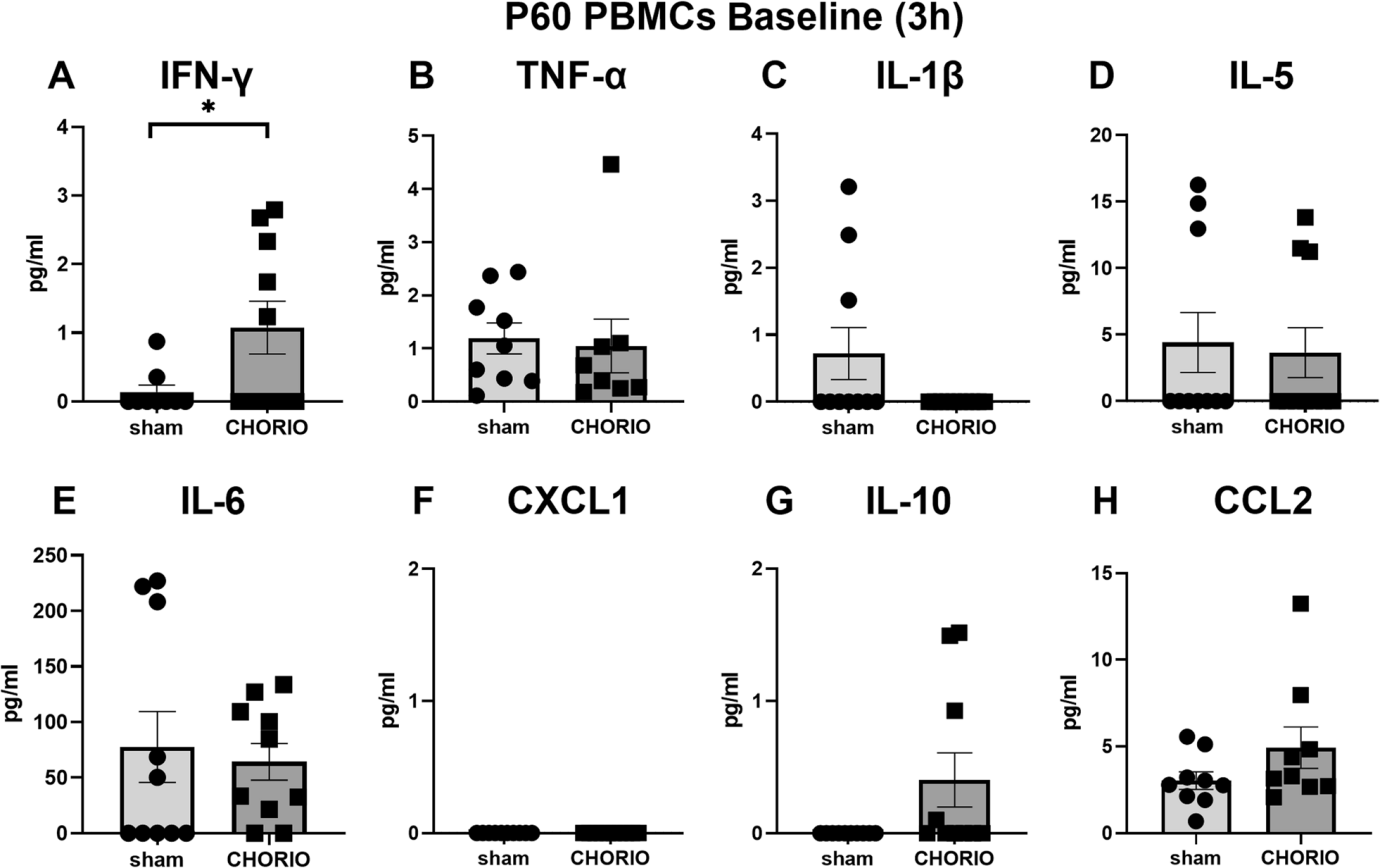
Cytokine and chemokine measurements at baseline in conditioned medium of PBMCs at P60 after 3 h in culture. PBMCs exposed to CHORIO in utero secreted significantly more IFN-γ compared to PBMCs from sham controls. Other cytokine and chemokine levels in the secretome at baseline did not differ. (^*^p < 0.05)

After assessing serum cytokines, we then examined PBMC reactivity at P60 (*n* = 10/group). At baseline, PBMCs from rats exposed to CHORIO in utero secreted significantly more IFN-γ after 3 h in culture compared to PBMCs from sham controls (sham: 0.14 ± 0.10 pg/ml, CHORIO: 1.08 ± 0.38 pg/ml, *t*-test, *p* < 0.05) (Fig. 3). No other significant increases were observed with other cytokines, IL-1β, IL-5, IL-6, IL-10, CXCL1, TNF-α, and CCL2 at baseline with either 3 h or 24 h in culture.

**Fig. 4:**
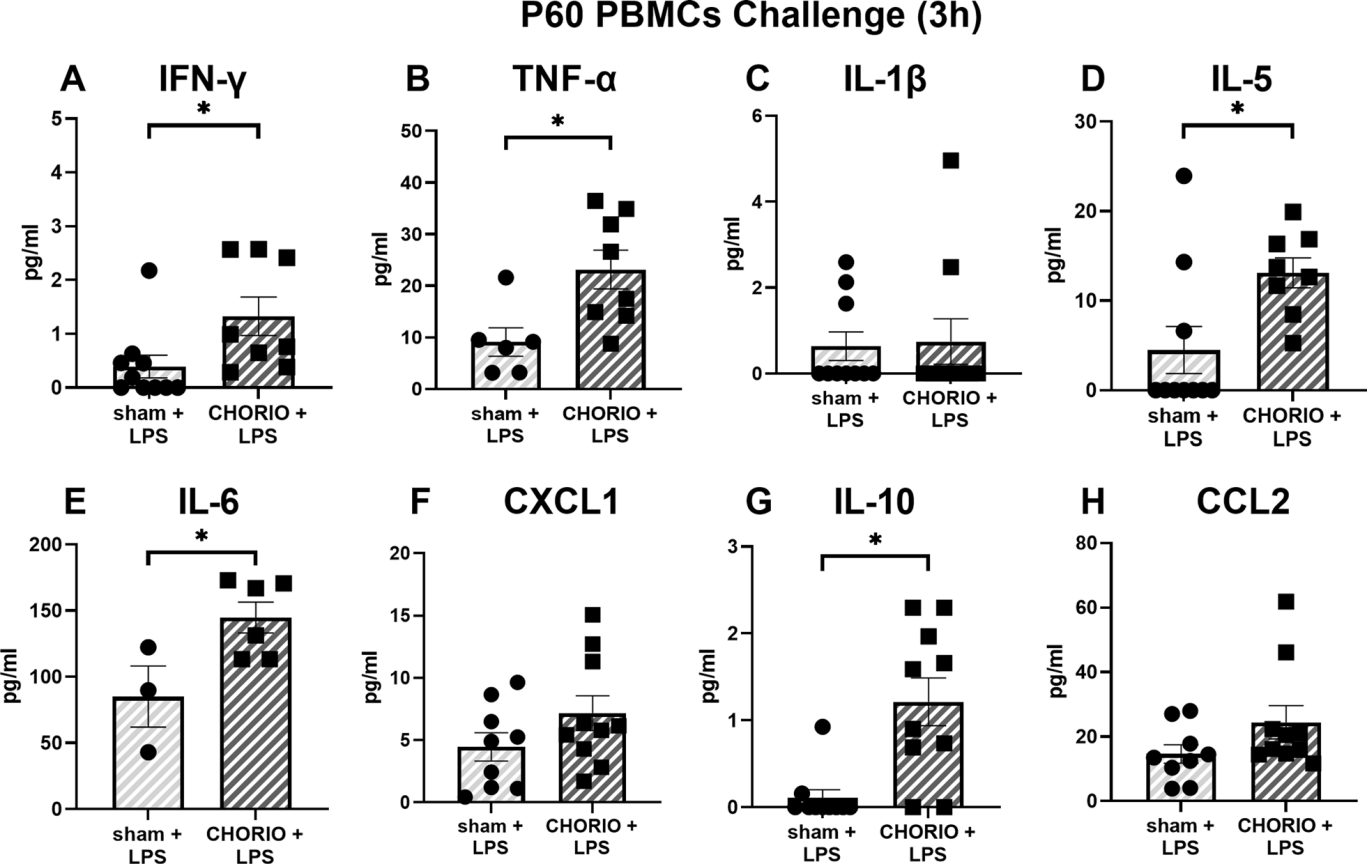
Response of PBMCs to secondary immune challenge with LPS stimulation at P60 after 3 h in culture. LPS stimulation significantly increased the levels of IFN-γ, TNF-α, IL-5, IL-6, and IL-10 in the secretome from CHORIO PBMCs compared to sham controls. (^*^p < 0.05)

**Fig. 5:**
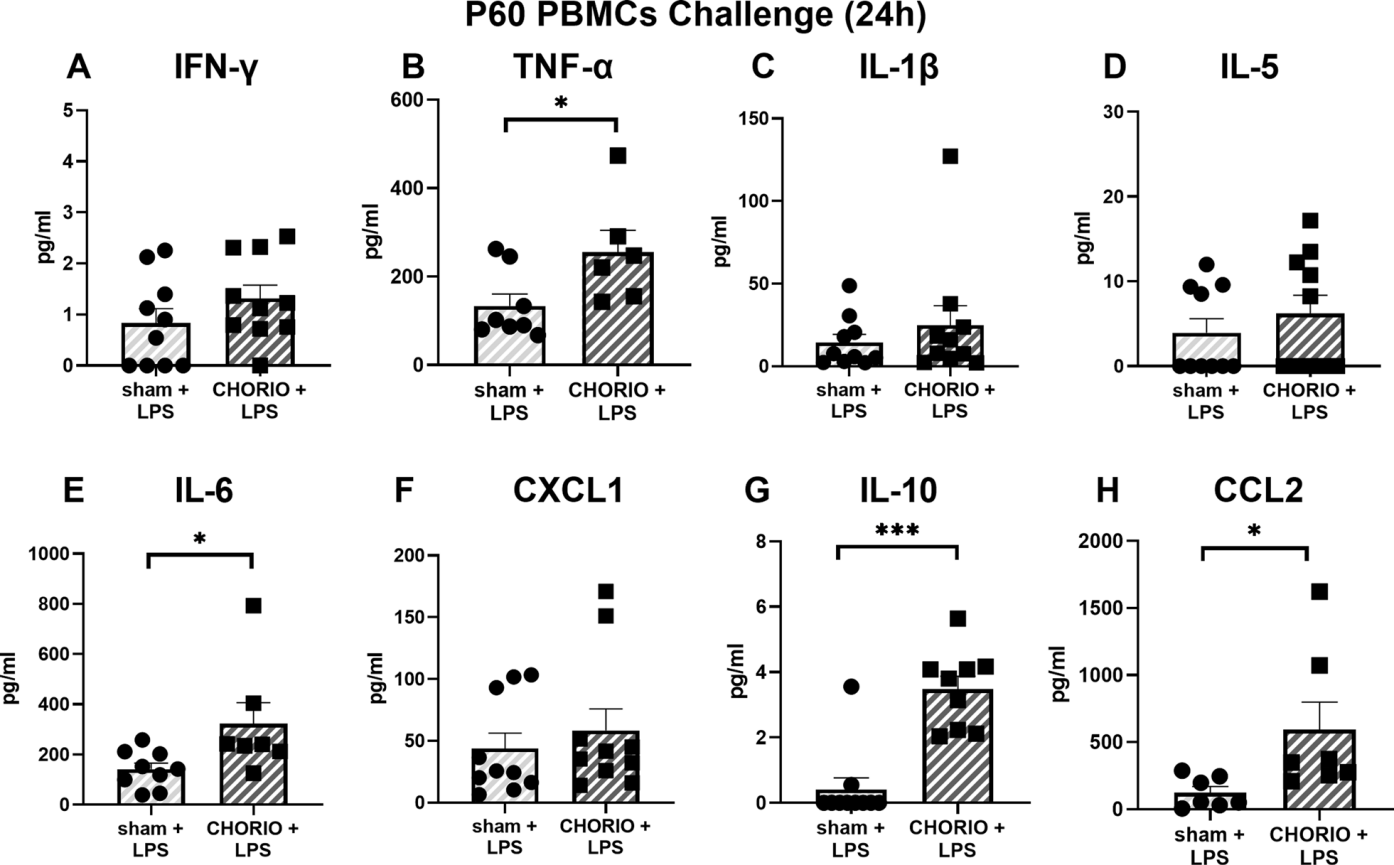
Response of PBMCs to secondary immune challenge with LPS stimulation at P60 after 24 h in culture. LPS stimulation significantly increased the secretion of TNF-α, IL-6, IL-10, and CCL2 in CHORIO PBMCs compared to sham controls. (^*^p < 0.05, ^***^p < 0.001)

After establishing baseline PBMC cytokine hypersecretion was specific to IFN-γ at P60, we next examined PBMC reactivity in response to a secondary immune challenge with LPS (*n* = 10/group). After both 3 h and 24 h in culture, LPS stimulation significantly increased TNF-α and IL-6 secretion into the CHORIO PBMC secretome compared to sham controls (Figs. 4 and 5). Specifically, TNF-α a secretion was increased by 2.5-fold at 3 h (Fig. 4B) and 1.9-fold at 24 h compared to sham (Fig. 5B). Similarly, IL-6 levels in the CHORIO secretome was 1.7-fold increased at 3 h (Fig. 4E) and 2.28-fold increase at 24 h (Fig. 5E) compared to sham. An effect with IL-5 and IFN-γ was also observed at 3 h (Fig. 4A, D). Specifically, IFN-γ was 2.3-fold increased at 3 h compared to sham (Fig. 4A). However, at 24 h, IL-10 and CCL2 levels in the secretome from LPS stimulated PBMCs were significantly different in CHORIO animals compared to sham (Fig. 5G, H). Indeed, IL-10 was 8.5-fold increased and CCL2 was increased 4.75-fold in the CHORIO secretome compared to sham. Taken together, these data reveal a robust SPIHR and protracted time course of cytokine secretion in stimulated PBMCs from CHORIO animals.

### In utero insults alter inflammatory responses at P120

**Fig. 6:**
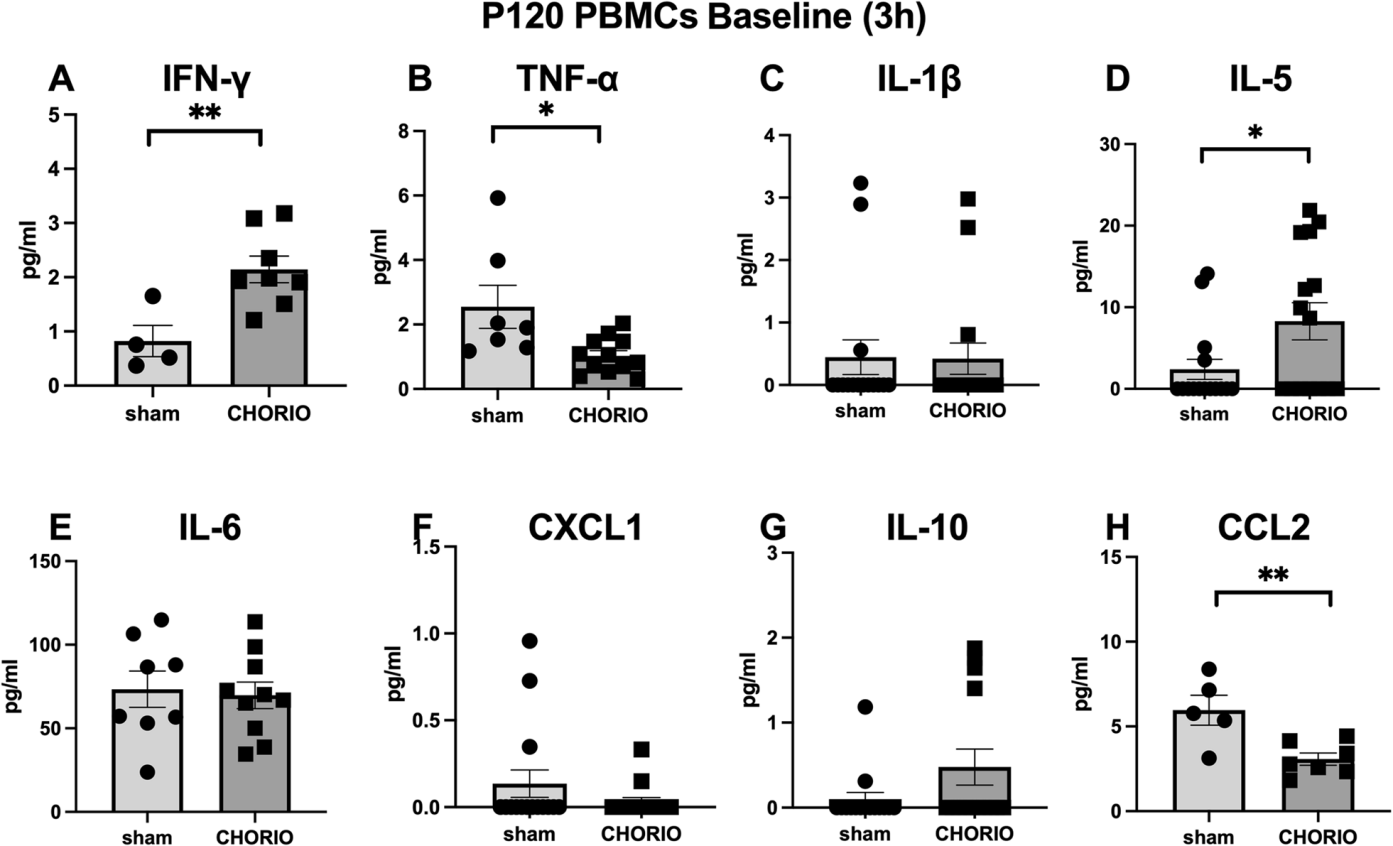
Cytokine and chemokine levels at baseline in the PBMC secretome at P120 after 3 h in culture. Levels of IFN-γ and IL-5 were higher in the secretome, while levels of TNF-α and CCL2 were lower, compared to sham controls at baseline. (^*^p < 0.05, ^**^p < 0.01)

**Fig. 7:**
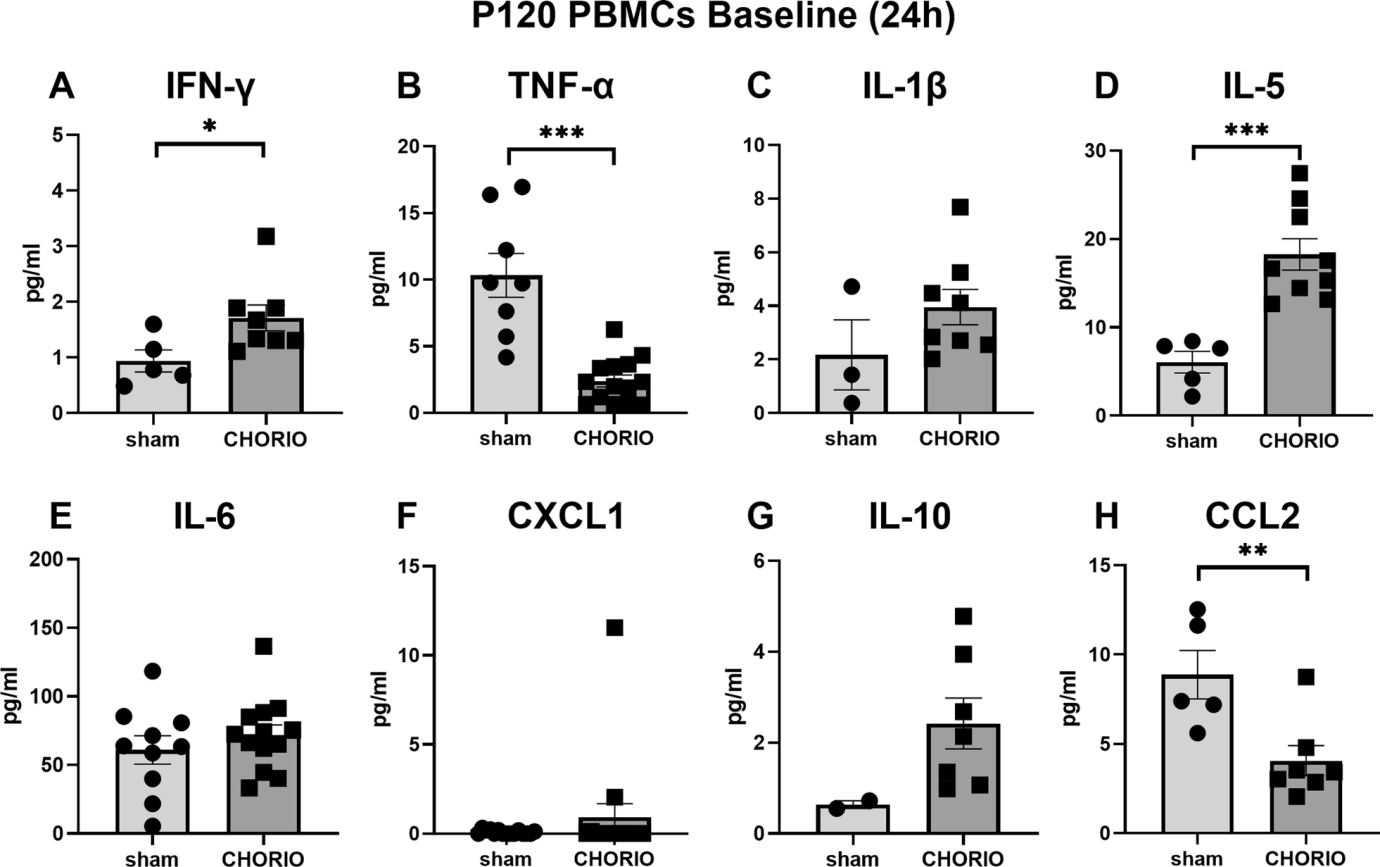
Cytokine and chemokine levels at baseline in the PBMC secretome at P120 after 24 h in culture. Similar to the pattern observed after 3 h, levels of IFN-γ and IL-5 were higher in the secretome at P120, while levels of TNF-α and CCL2 were lower, compared to sham controls at baseline (^*^p < 0.05, ^**^p < 0.01, ^***^p < 0.001)

After confirming the changes in peripheral inflammatory reactivity associated with perinatal injury at P60, PBMCs were isolated and stimulated at P120 (*n* = 13/group). We evaluated secreted cytokine levels at this older adult age to further understand immune signatures of in utero injury throughout the lifespan. In unstimulated PBMCs at P120, PBMCs isolated from CHORIO rats showed significantly higher baseline IFN-γ secretion compared to sham after 3 h in culture (sham: 0.82 ± 0.29 pg/ml, CHORIO: 2.14 ± 0.25 pg/ml, *t*-test, *p* < 0.01), and after 24 h (sham: 0.94 ± 0.20 pg/ml, CHORIO: 1.71 ± 0.23 pg/ml, *t*-test, *p* < 0.05) (Figs. 6 and 7). IL-5 levels in the PBMC secretome at baseline was also increased at baseline (sham: 2.39 ± 1.24 pg/ml, CHORIO: 8.29 ± 2.3 pg/ml, *t*-test, *p* = 0.03) (Fig. 6D). The secretion level of IL-5 in PBMCs of CHORIO at 24 h was significantly higher than that of sham (sham: 6.07 ± 1.22 pg/ml, CHORIO: 18.3 ± 1.8 pg/ml, *t*-test, *p* < 0.001) (Fig. 7D). Interestingly, the baseline secretion of TNF-α and CCL2 from PBMCs was lower in CHORIO rats than in sham after 3 h and 24 h. Specifically, TNF-α was decreased by 40.4% at 3 h and decreased by 23.3% at 24 h (Fig. 6B, *t*-test, *p* < 0.01; Fig. 7B, *t*-test, *p* < 0.0001). Similarly, CCL2 baseline secretion was decreased by 51.7% at 3 h and decreased by 45.8% at 24 h in CHORIO PBMCs compared to sham (Fig. 6H, *t*-test, *p* < 0.01; Fig. 7H, *t*-test, *p* < 0.01).

**Fig. 8:**
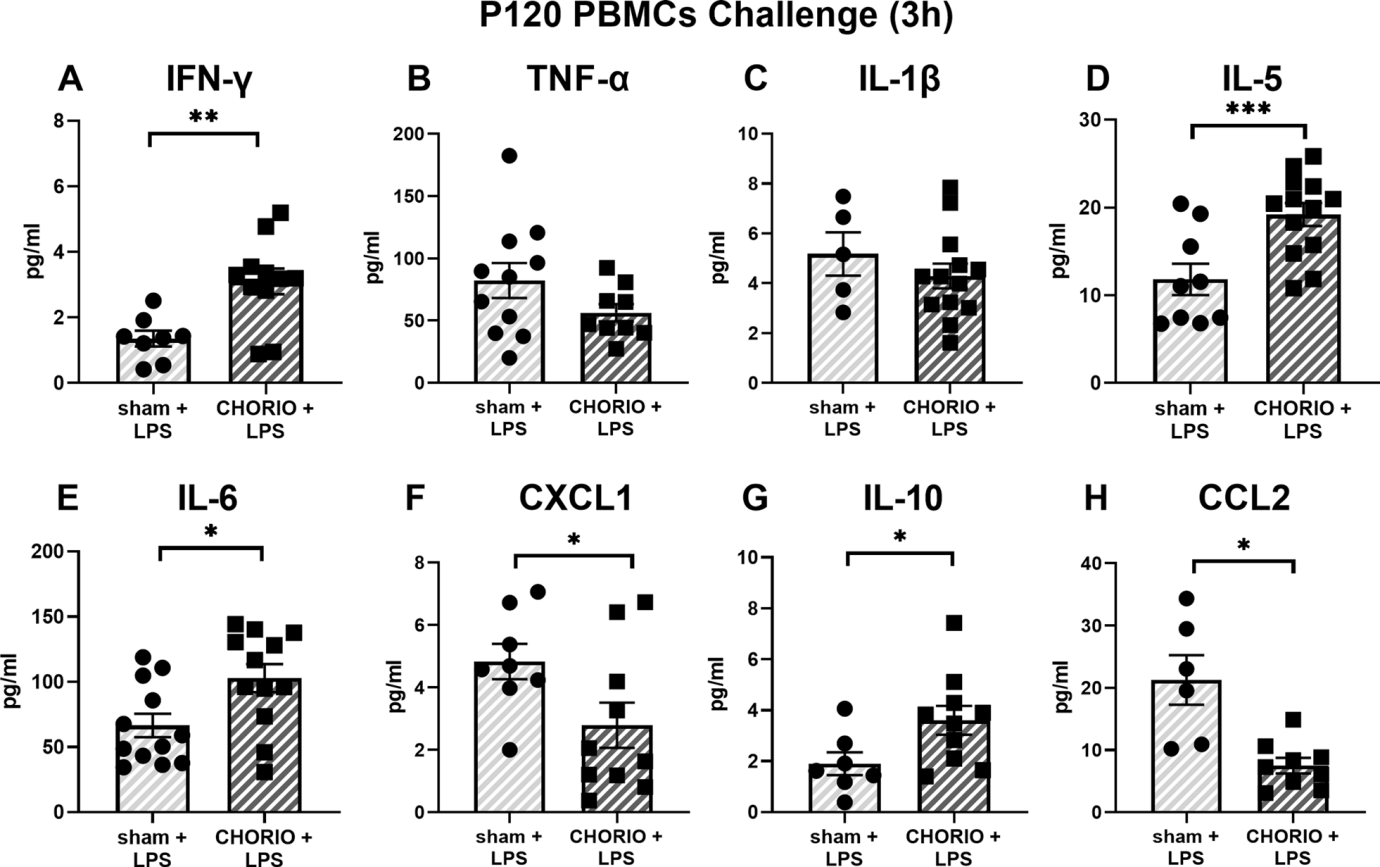
Response of PBMCs after secondary immune challenge with LPS stimulation at P120 after 3 h in culture. The levels of IFN-γ, IL-5, IL-6, and IL-10 was significantly higher in the secretome from CHORIO PBMCs compared to sham, while levels of CXCL1 and CCL2 was significantly reduced in CHORIO PBMCs compared to sham (^*^p < 0.05, ^**^p < 0.01, ^***^p < 0.001)

**Fig. 9:**
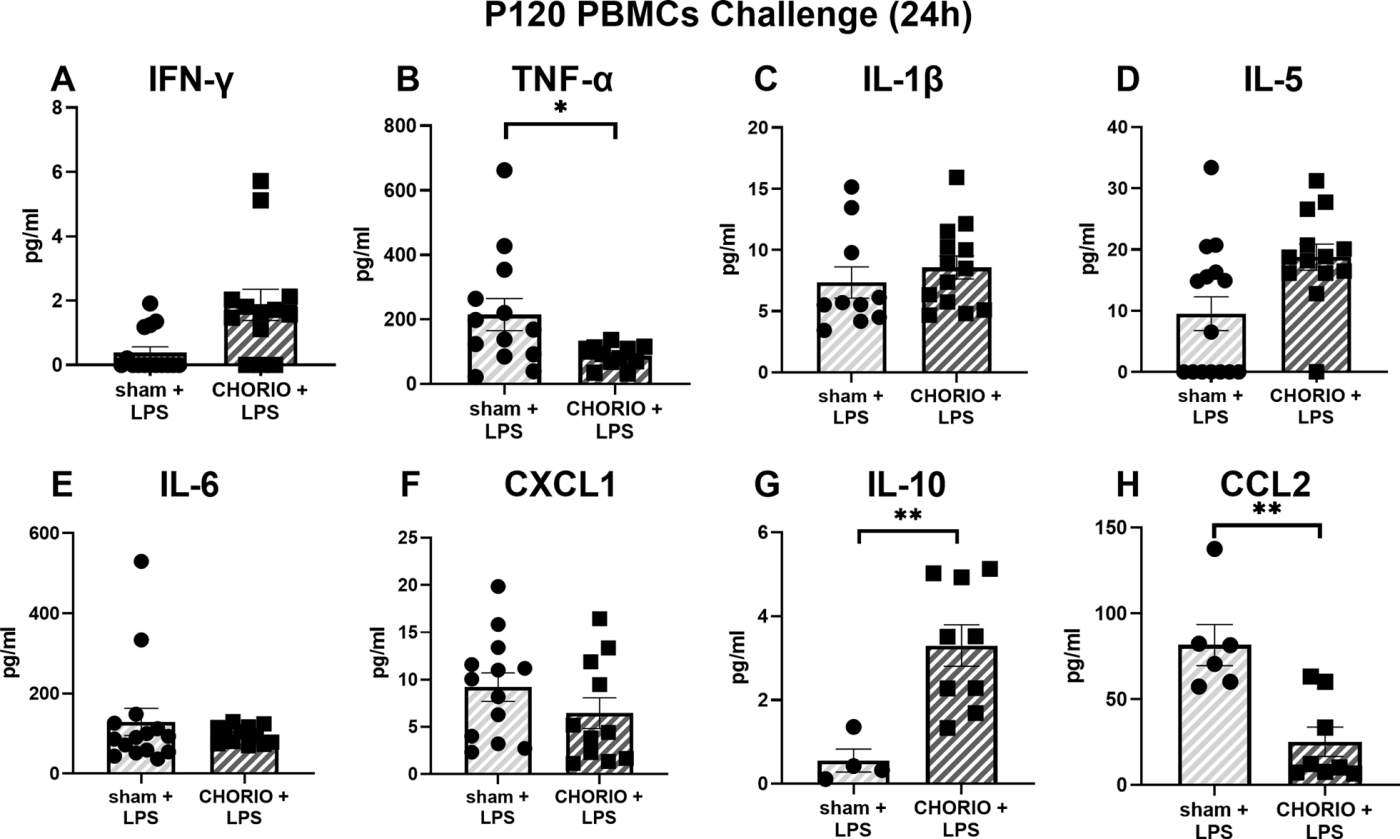
Response of PBMCs after secondary immune challenge with LPS stimulation at P120 after 24 h in culture. Levels of IL-10 in the secretome were significantly higher in CHORIO compared to sham, while levels of TNF-α and CCL2 in the secretome were significantly decreased in CHORIO compared with sham. (^*^p < 0.05, ^**^p < 0.01)

We next evaluated the level of cytokines in the PBMC secretome after LPS challenge (*n* = 13/group). At 3 h after LPS stimulation, IFN-γ was increased by 2.3-fold in the CHORIO PBMC secretome compared with sham (Fig. 8A, *t*-test, *p* < 0.01). The secretion of CHORIO in IL-5 was increased 1.6-fold (Fig. 8D, *t*-test, *p* < 0.001), IL-6 was increased 1.5-fold (Fig. 8E, *t*-test, *p* < 0.05) and IL-10 was elevated 1.9-fold (Fig. 8G, *t*-test, *p* < 0.05) in the CHORIO PBMC secretome compared with sham. At 24 h after LPS challenge, IL-10 increased by 3.25-fold (Fig. 9G, *t*-test, *p* < 0.01) compared with shams.

In contrast to these increases, CXCL1 was decreased by 57.7% (Fig. 8F, *t*-test, *p* < 0.05) and CCL2 was decreased by 35.3% Fig. 8H, *t*-test, *p* < 0.01) in the CHORIO secretome compared with the sham secretome 3 h after LPS challenge. By 24 h, TNF-α was reduced by 40.6% (Fig. 9B, *t*-test, *p* < 0.05) and CCL2 was decreased by 30.8% (Fig. 9H, *t*- test, *p* < 0.01**)** compared with sham.

### In utero injury induces long-term changes in peripheral and central immune cell populations

**Fig. 10:**
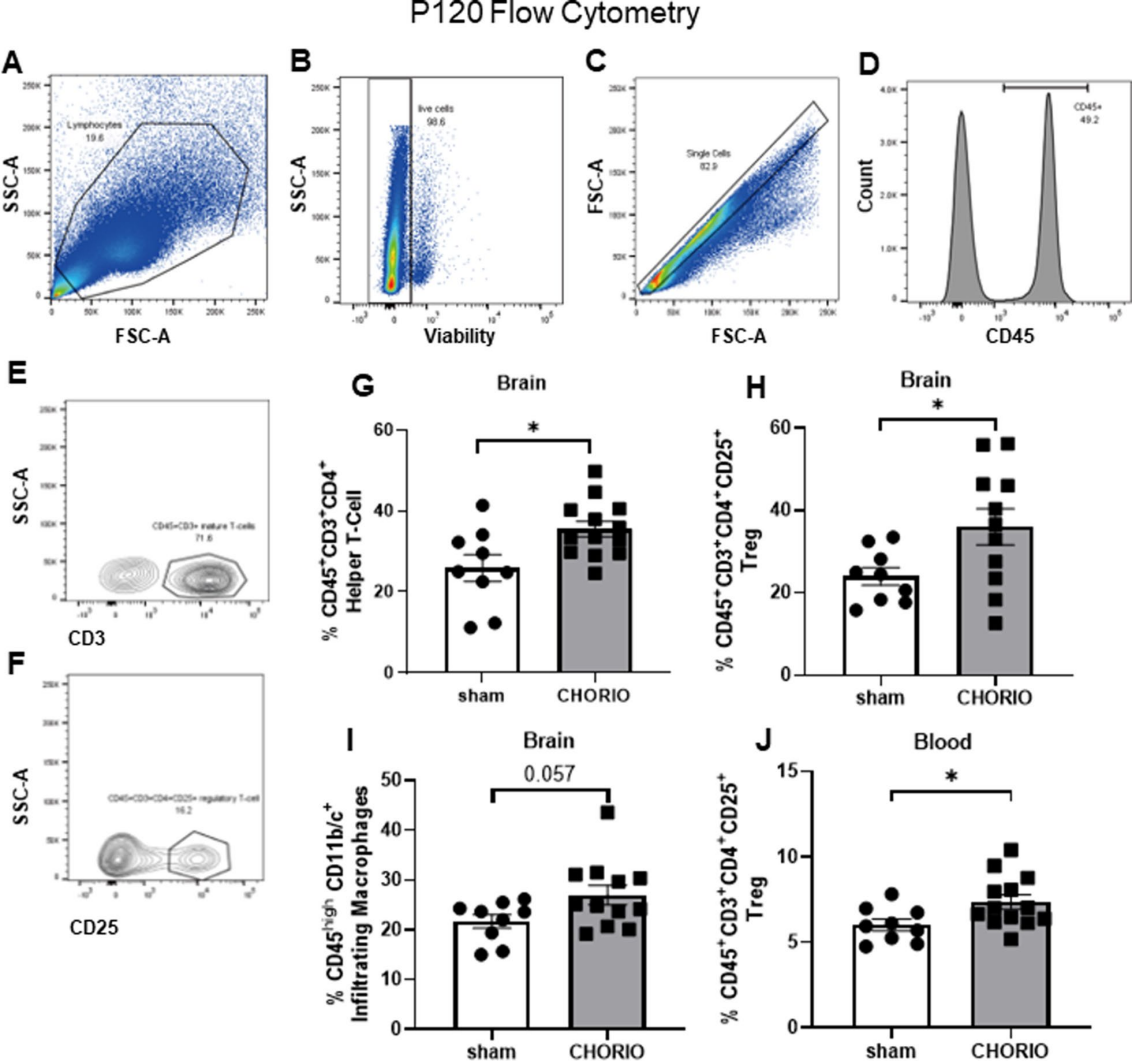
Flow cytometry analysis was used to identify proportions of myeloid cells at P120 and reveals distinct changes in T cells in CHORIO rats. Notably, proportions of viable (identified by size, granularity and viability dye staining, **A**–**D**) T helper cells (CD45^+^CD3^+^CD4^+^) in the brain (**G**), and T regulatory cells (CD45^+^CD3^+^ CD4^+^CD25^+^) in the brain (**H**) and blood (**J**) are increased in CHORIO rats compared to sham controls. Similarly, the proportion of cells with positive expression for infiltrating macrophage markers (CD45^high^CD11b^+^) are also increased in the brain of CHORIO rats at P120 compared to sham (**I**). Gating for CD3^+^ and CD25^+^ cells is shown in **E** and **F** (^*^p < 0.05, *FSC*  forward scatter, *SSC*  side scatter). (Data represent at least 3 independent experiments.)

To investigate the long-term inflammatory cell population changes in the brain and periphery persisting after CHORIO, multiparameter flow cytometry was performed at P120 (sham *n* = 9, CHORIO *n* = 13). Cells were gated first on size and granularity (Fig. 10A), followed by gating on live cells (verified by viability dye staining) (Fig. 10B) then single cells (Fig. 10C). Next, CD45-positive cells were identified (Fig. 10D) [74, 75]. To identify monocyte, macrophages, and neutrophils, CD45^+^ cells were then analyzed for CD11b/c expression. CD45^+^ CD11b/c^+^ cells were further gated for Ly6G expression to separate neutrophils. In the brain, CD45^+^ CD11b/c^+^ cells were further subdivided based on CD45 expression. CD45^high^CD11b/c^+^ cells were considered monocytes/macrophages, while resident microglia were characterized as CD45^low/med^CD11b/c^+^ as previously described [74–76]. In a separate panel, T cells were identified based on CD45^+^ CD3^+^ staining (Fig. 10E). Consistent with prior literature, CD45^+^ CD3^+^ CD4^+^ were considered Helper T cells and CD45^+^ CD3^+^ CD4^+^ CD25^+^ were considered regulatory T cells (Tregs) [77, 78] (Fig. 10F).

Results of these flow cytometric assays reveal unique immune population shifts at P120, greater than 4 months after in utero insult. While no significant differences in neutrophils were observed in either blood or brain between sham and CHORIO rats, a significant increase in helper T cells (CD45^+^ CD3^+^ CD4^+^) (Fig. 10G, *t*-test, *p* < 0.05), and regulatory T cells (CD45^+^ CD3^+^ CD4^+^ CD25^+^) (Fig. 10H, *t*-test, *p* < 0.05) in the brains of CHORIO rats were observed. Additionally, there was a trend towards increased CD45^+^CD11b/c^+^-positive cells (Fig. 10I, *t*-test, *p* = 0.057**)** in CHORIO brains compared to sham controls. Analyses of the blood confirmed an increase in circulating regulatory T cells (CD45^+^ CD3^+^ CD4^+^ CD25^+^) in CHORIO animals compared to sham (Fig. 10J, *t*-test, *p* < 0.05**)**.

## Discussion

In this study, we provide the first evidence that the immune system is chronically hyper-reactive and primed to an immune challenge later in life secondary to chorioamnionitis*.* In the context of this preclinical model of CP, we specifically demonstrate that exposure to prenatal inflammation leads to SPIHR and immune response dysfunction that persists well into adulthood. These changes are concomitant with hypercytokinemia, including elevated serum IL-1β, TNF-α, IL-6, CCL2 and CXCL1 through P60 and altered T cell population dynamics in both the brain and peripheral circulation at P120. Indeed, P120 is equivalent to a rat well into middle age and represents a time point over 4 months following exposure to CHORIO. We also found changes in regulatory T cell and helper T cell populations at P120 with hypersecretion of IFN-γ, CCL2, IL-6 and TNF-α, known macrophage and monocyte traffickers. Specifically, TNF-α and IL-1β are both produced by cells throughout the CNS, and IL-6 is required for cellular responses located on the surface of microglia [79, 80]. IL-6 also strongly induces CCL2 mRNA expression and secretion of CCL2 by PBMCs [81]. CCL2 is a cytokine that mobilizes monocytes and T cells to sites of inflammation [82, 83]. The unique signature of inflammatory cytokine and chemokine expression documented here, and the reactivity and enduring PBMC response in this translational model, may be a potential biomarker for assessing inflammation during the perinatal period, perinatal brain injury and neurodevelopmental disorders including CP. Indeed, baseline PBMC reactivity, coupled with subsequent LPS stimulation, reveals unique signatures of cellular inflammation that serum cytokine measurements do not capture. These collection and culture protocols are robust and reproducible, and future studies of PBMC reactivity could be implemented in adults with CP to guide rehabilitative and emerging therapeutic approaches. Additionally, PBMC profiling may prove useful in defining risk of later life inflammatory sequelae in children with CP, including alterations in the trajectory of myelination, and the development of chronic pain.

Neural–immune cross talk is an essential component of health and physiology but can become maladaptive, especially in a brain primed by pre-existing inflammation or prior insult [84]. Immune plasticity altered after prenatal inflammatory priming or programing may have long-term effects on the responses and function of circulating leukocytes [58]. Notably, neonates are sensitive to programming that permanently affects development and preterm infants are susceptible to inflammatory disorders [58]. Here, we confirm that the cerebral and peripheral microenvironments are profoundly inflammatory well into adulthood after CHORIO and that circulating leukocytes are abnormal over the lifespan. Not only are serum cytokine levels high, but immune cells are also hyper-reactive to secondary immune challenge leading to release of additional pro-inflammatory mediators including IFN-γ, TNF-α, IL-6 and CCL2. The persistent presence of these soluble inflammatory mediators is capable of not only disrupting the blood–brain barrier directly, but also facilitating the migration and trafficking of macrophages, monocytes and neutrophils. Additional cytokines and chemokines produced by these circulating leukocytes can move freely across the blood–brain barrier, impair its function and further increase immune cell access to brain parenchyma. This compounds the activation and stimulation of resident glia, and propagates neuroinflammation and a toxic cerebral microenvironment. In this context, multiple overlapping mechanisms can then contribute to brain injury, including direct initiation of programmed cell death pathways in neurons and oligodendrocytes, microglial and astrocyte activation, mitochondrial damage and endoplasmic reticulum stress [85–87]. Persistent hypercytokinemia can also lead to inhibition of cortical branching, dysfunctional synapse formation and pruning, and propagation of white matter injury [88, 89]. Cumulatively, this leads to profound structural and functional changes, aberrant connectivity and disrupted neural networks leading to lasting neurological sequelae [9, 56, 63, 64, 72, 84, 90, 91]. This is important in the context of preterm infants and former preterm infants where anatomical, functional, and local connectivity is known to be impaired [92, 93]. Specifically, preterm infants have fragmented functional networks resulting in less integration, reduced intra-hemispheric connectivity yielding less transmission capacity, and less global efficiency compared to term infants; these changes impact brain function through the lifespan [94–96].

Here, we found increased levels of regulatory T cells and helper T cells that persisted over four months after an in utero insult consistent with lack of lymphocyte homeostasis and potential compensatory response to ongoing inflammation. Although the excessive production of inflammatory molecules and activation of immune cells in the acute phase of perinatal brain injury can lead to loss of BBB integrity and lymphocyte infiltration injury [48, 49], the enduring consequences of chronic immune population shifts and hyper-reactive PBMCs are not well understood. Indeed, phenotypic plasticity of lymphocytes may be maladaptive and important to injury progression, or at the very least, a hindrance to neural repair in many individuals with CP or other forms of perinatal brain injury. Similar findings have been documented in children with Down Syndrome [97, 98] and after exposure to opioids [59, 70]. On the other hand, decreased levels of pro-inflammatory cytokines in sera and PBMC supernatants have been found in response to LPS stimulation in critically ill patients in intensive care [99, 100], and in children with neonatal encephalopathy who received hypothermia [100, 101]. Hyporeactivity to LPS stimulation has also been observed in children with CP when IL-2, IL-6, IL-1α and IL-1β were studied [100]. As has been eloquently described by other investigators, LPS is not only capable of inducing a cascade of inflammatory pathways but also initiates a refractory state, known as LPS tolerance [100, 102, 103]. This state is associated with a decreased capacity of blood cells and PBMCs to produce pro-inflammatory cytokines in response to stimulation [100]. At P120 we found that lower levels of CCL2, CXCL1 and TNF-α were secreted by PBMCs from CHORIO animals after LPS challenge. This LPS tolerance exists in the context of increased T-helper and T regulatory cells and previous LPS hyper-reactivity. Together, these data suggest that the function of T cells themselves may also be altered. With advancing age, the number of T regulatory cells increases and their composition changes [104], and an association between increased number of T regulatory cells, chronically activated microglia, choroid plexus secretion of toxic versus trophic factors and the development of neurodegenerative diseases has been reported [105]. In addition, when the inducibility and durability of regulatory T cell responses change, there is inevitably an imbalance between protective and pathogenic immunity, which may sustain, chronic tissue-damaging inflammation [104]. Notably, the differentiation, survival, and regulation of T regulatory cells depend on complex interactions with cytokines, and conversely, the regulation of pro-inflammatory cytokines such as IFN-γ is also critically dependent on T regulatory cells [106]. Considering the high reactivity of PBMCs observed after CHORIO, the subclassification of cytokines persistently detected, and the changes in the number of T regulatory cells through P120, it is possible that changes in regulatory T cell function and population dynamics may feed forward white matter injury and neuronal dysfunction with aging or hinder neural cell repair. Taken together, these data emphasize dynamic immune modulation with maturation and injury evolution, the complex neural–immune pathology secondary to chorioamnionitis, and the functional consequences of a persistently toxic cerebral microenvironment.

As stated above, our data support that CHORIO causes persistent changes in both the peripheral and neuroinflammatory microenvironment with enduring immune cell and molecular alterations throughout the lifespan. Interestingly, systemic inflammation has been proposed to be a final common denominator for insults caused by both hypoxia–ischemia and infection leading to brain injury in infants [88, 107–109]. We utilized an established rat model of CHORIO that yields cerebral palsy-like deficits in the mature CNS that mimic those of preterm survivors, with a spastic-like gait, poor social interaction, and cognitive impairment [59, 61, 62, 71]. This model encompasses the FIRS, SIRS and placental pathology common in preterm infants with CHORIO [62], including acutely elevated IL-1β, TNF-α, IL-6, IL-10, and CXCL1 in serum [59, 62, 64, 110]. Reflecting the significant inflammation transduced through the placental–fetal brain axis, rats with CHORIO grow up to have complex gait abnormalities, cognitive and executive function abnormalities and white matter injury in adulthood [59–61, 64, 111]. The systemic inflammation associated with CHORIO causes PBMC hyper-reactivity at P7 (term-equivalent human age) and P21 (toddler-equivalent human age) [59]. Importantly, these findings are extended here and confirm that immune dysregulation and PBMC hyper-reactivity is present in adulthood, and defined by increased levels of IFN-γ, CCL2, IL-6 and TNF-α in the PBMC secretome. This is significant as the inflammation observed following perinatal brain injury appears to feed forward unchecked through much of the lifespan. Indeed, levels of pro-inflammatory proteins in the PBMC secretome are increased at baseline in rats with in utero injury even without exposure to immune challenge or secondary LPS exposure.

These data corroborate findings and conclusions from other groups who have studied CP [58, 85, 100], including increased PBMC reactivity in former preterm children with CP in school age and teenagers with CP. Collectively, these results provide additional support for the hypothesis that persistent inflammation may prevent regeneration or exacerbate brain injury in patients with CP and sensitize the brain to further or additional injury encompassing a tertiary phase of CP pathophysiology [85]. While brain injury may be propagated by persistent inflammation through epigenetic changes, the direct impact on maturation and differentiation of oligodendrocyte subpopulations, impaired axonal growth and dynamic myelination, and effects on the neurogenic niche caused by sustained expression cytokines, primed immune cells, and aberrant immune cell trafficking cannot be overemphasized. Our data showing persistent hypercytokinemia, robust SPIHR, and multiple chronic shifts in immune cell populations through P120 in the rat support the concept that the tertiary phase of perinatal brain injury and CP may last for months (in rats) or years (in humans) after the initiating insult [85]. Together with our prior data confirming cognitive and executive function deficits in adult animals in this model of CP, the negative impact of ongoing inflammation in the trajectory of brain development, including cognitive and motor performance is reaffirmed [59, 61, 112–114]. This has been validated in other models of brain injury, including traumatic brain injury, where microglial activation and immune cell dysfunction has been shown to last for upwards of 10 years following the initiating insult concomitant with poor cognitive function [115]. In experimental sepsis, persistent cognitive impairment has been observed in rats that have completely recovered clinically with associated negative blood cultures [84, 116]. This indicates that resolution of the inflammatory trigger (i.e., resolution of CHORIO, FIRS, or SIRS), does not prevent ongoing brain injury [84]. Clinical studies in children with CP confirm altered inflammatory responses that persist for at least 6–14 years [58, 100]. As increased peripheral cytokines are a marker of cerebral inflammation, the time course for altered inflammatory responses in CP is likely to last for decades. Undoubtedly, future studies focused on PBMC reactivity in older patients with CP, including beyond the teenage years would be very valuable to the field, as would studies using immunomodulatory treatment to enable long-term recovery and regeneration.

This study is not without limitations and is constrained by scope. First, our study was not powered to detect or exclude sex differences despite both sexes being used in every experiment and all outcome measures. Further investigation beyond the scope of the present investigation will examine sex differences in PBMC priming and reactivity throughout the lifespan. This is an essential next step as evidence from studies examining PBMCs from adult humans suggest sex differences in stimulated PBMC properties and secretion exist [117–119]. Second, we were unable to define specific mechanisms responsible for persistent changes in immune reactivity. Future studies of mechanism and immune function will be important for revealing potentially novel therapeutic targets to prevent neural–immune injury secondary to CHORIO and for understanding the consequences of immune changes to subsequent challenges, insults and injuries through the lifespan. Although, we cannot do serial sampling of cytokines and PBMC reactivity assessments longitudinally in the same animal, and there are inherent limitations of working with rodents. PMBCs represent a heterogeneous population of peripheral circulating mononuclear cells consisting of T cells, T-regulatory cells, T-helper cells, B cells, and natural killer/ dendritic cells/ monocytes [120]. Additional flow cytometric studies at discrete time points with expanded panels, are needed to clarify the exact composition of the immune cell populations in our models. Similarly, it would be interesting in future studies to understand the contributions of other cell types to the inflammatory secretome present in isolated whole blood longitudinally.

## Conclusion

In conclusion, we found significant, dynamic, and chronic inflammatory responses in adult rats months after exposure to CHORIO. In addition to upregulation of inflammatory biomarkers in serum through adulthood, we also demonstrated hyper-responsiveness to LPS stimulation at P60, contrasted by hypo-responsiveness to LPS at P120. These dynamic inflammatory changes and altered cytokine responses are consistent with a chronically modulated neural–immune microenvironment, persistent inflammatory state and maladaptive lymphocyte plasticity. Taken together, these data shed light on mechanisms of ongoing brain injury in individuals with CP and emphasize the need for novel liquid diagnostic biomarkers to track the degree of injury, and response to potential interventions. Indeed, the durable changes in PBMCs and their responses to subsequent immune stimuli may function as a biomarker useful for indirectly evaluating brain damage or detecting sustained neural–immune injury during the perinatal period and later in life. Similarly, these data underscore the role of immunomodulatory therapy in the treatment of CP and preterm infants exposed to CHORIO. Together, with a growing body of preclinical and clinical data, our results highlight the importance of long-term outcome measures in future clinical trials for preterm infants and the need for long-term follow-up in patients with brain injury in the NICU.

## Data Availability

All data generated and analyzed during this study are available from the corresponding author on reasonable request.

## Abbreviations

ARRIVE: Animals in research reporting in vivo experiments
BBB: Blood–brain barrier
BPD: Bronchopulmonary dysplasia
CCL2: C–C motif chemokine ligand 2
CD: Cluster of differentiation
CHORIO: Chorioamnionitis
CNS: Central nervous system
CP: Cerebral palsy
CXCL1: C–X–C motif chemokine ligand 1
E: Embryonic
FBS: Fetal bovine serum
FIRS: Fetal inflammatory response syndrome
IFN-γ: Interferon gamma
IL: Interleukin
IVH: Intraventricular hemorrhage
LPS: Lipopolysaccharide
Ly6G: Anti-lymphocyte antigen 6G
MCP-1: Monocyte chemoattractant protein-1
MECI: Multiplex electrochemiluminescent immunoassay
mRNA: Messenger ribonucleic acid
NEC: Necrotizing enterocolitis
NICU: Neonatal intensive care unit
P: Postnatal
PBI: Perinatal brain injury
PBMCs: Peripheral blood mononuclear cells
RPMI: Roswell Park Memorial Institute
SIRS: Systemic inflammatory response syndrome
SPIHR: Sustained peripheral immune hyper-reactivity
TNF: Tumor necrosis factor
